# Placoderm Assemblage from the Tetrapod-Bearing Locality of Strud (Belgium, Upper Famennian) Provides Evidence for a Fish Nursery

**DOI:** 10.1371/journal.pone.0161540

**Published:** 2016-08-23

**Authors:** Sébastien Olive, Gaël Clément, Edward B. Daeschler, Vincent Dupret

**Affiliations:** 1 OD Earth and Life History, Royal Belgian Institute of Natural Sciences, Brussels, Belgium; 2 Evolution & Diversity Dynamics Lab, Department of Geology, Liège University, Liège, Belgium; 3 Centre de Recherche sur la Paléobiodiversité et les Paléoenvironnements, UMR CR2P 7207, Sorbonne Universités, MNHN, CNRS, UPMC-Paris6, Muséum national d’Histoire Naturelle, Paris, France; 4 Academy of Natural Sciences of Drexel University, Philadelphia, Pennsylvania, United States of America; 5 Subdepartment of Evolution and Development, Department of Organismal Biology, Uppsala University, Uppsala, Sweden; Raymond M. Alf Museum of Paleontology, UNITED STATES

## Abstract

The placoderm fauna of the upper Famennian tetrapod-bearing locality of Strud, Belgium, includes the antiarch *Grossilepis rikiki*, the arthrodire groenlandaspidid *Turrisaspis strudensis* and the phyllolepidid *Phyllolepis undulata*. Based on morphological and morphometric evidence, the placoderm specimens from Strud are predominantly recognised as immature specimens and this locality as representing a placoderm nursery. The Strud depositional environment corresponds to a channel in an alluvial plain, and the presence of a nursery in such environment could have provided nutrients and protection to the placoderm offspring. This represents one of the earliest pieces of evidence for this sort of habitat partitioning in vertebrate history, with adults living more distantly from the nursery and using the nursery only to spawn or give live birth.

## Introduction

The Strud quarry (Namur Province, Belgium) is one of the few localities in the world that has yielded Devonian tetrapod remains [1]. The tetrapod remains were found in association with very abundant flora [2], a putative insect [3–5], continental crustaceans [6–9] as well as sarcopterygian [10–12] and as yet undescribed acanthodian and actinopterygian fishes. The placoderm assemblage has been recently described and includes the antiarch *Grossilepis rikiki*, the groenlandaspidid *Turrisaspis strudensis* and the phyllolepidid *Phyllolepis undulata* [13,14].

In modern ecosystems, a nursery is defined as an area nearly exclusively inhabited by immature individuals [15]. A few Upper Palaeozoic localities, such as the Carboniferous chondrichthyan locality of Mazon Creek, USA [16] or the Famennian coelacanth locality of Waterloo Farm, South Africa [17], were described as nurseries. Others were hypothesized as such because they are dominated by immature individuals [18]. Carr [19] suggested the presence of a nursery from the Devonian of Merriganowry, Australia, based on a complete range of ontogenetic stages of the placoderm *Cowralepis*, but he did not mention any ratio of immature to adult forms. He also suggested the presence of a placoderm nursery in the Cleveland Shales (Famennian, Ohio), based on the discovery of egg cases, but to our knowledge this hypothesis has never been tested. Consequently, both localities constitute uncertain records of placoderm nurseries in the Devonian. Other localities, such as the Frasnian localities of Miguasha, Canada [20] and Lode, Latvia [21,22], include immature specimens in large numbers. However, because these localities display all ontogenetic stages, and not nearly exclusively immature individuals, they cannot be considered as nursery sites. A more convincing record is provided by the Upper Devonian locality of Tioga County, Pennsylvania, in which some assemblages are dominated by very young *Bothriolepis* specimens [23].

This article characterizes the ontogenetic composition of the placoderm material from Strud and considers the ecological implications for placoderms in this unique Upper Devonian site, interpreted here as a placoderm nursery.

## Material and Methods

The fossiliferous strata of the Strud quarry belong to the Upper Devonian Evieux Formation and are late Famennian in age [24]. Placoderm remains are found throughout lithologic unit 7, with the exception of its uppermost part [13].

**Fig 1:**
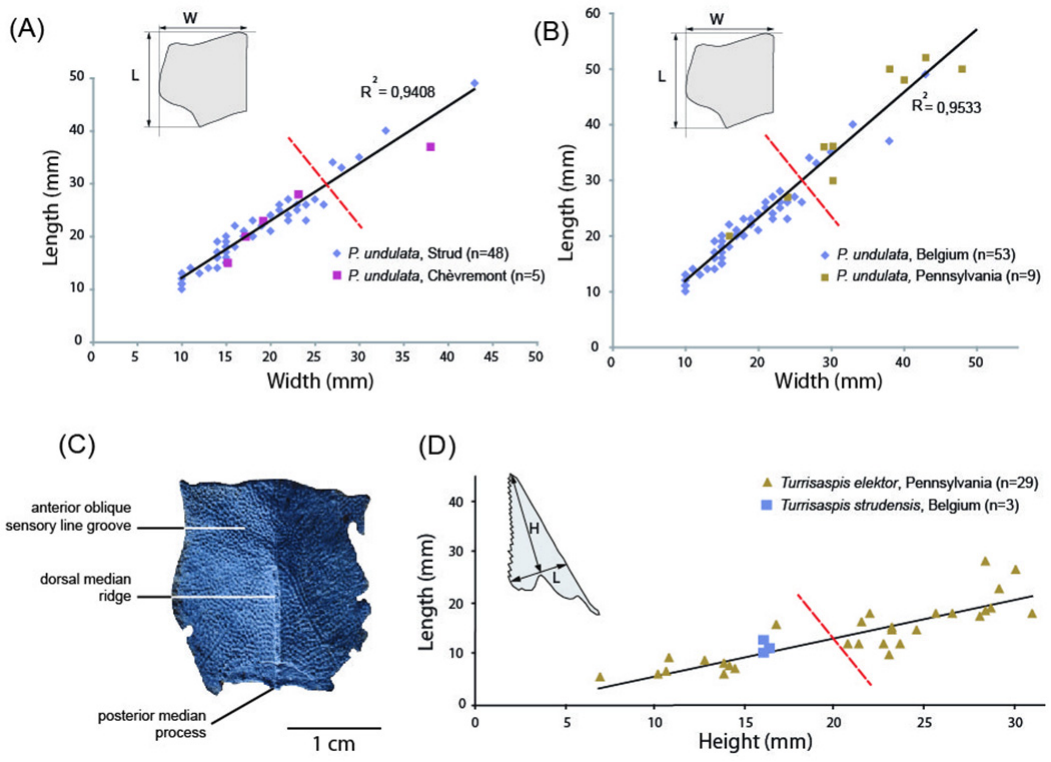
Graphical and anatomical elements characterizing the immature nature of the Strud placoderm material. (A) length vs. width graph of anterior ventrolateral plates of *Phyllolepis undulata* from Belgium. (B) length vs. width graph of anterior ventrolateral plates of *Phyllolepis undulata* from Belgium and Pennsylvania (number of specimens from Pennsylvania non-statistically valid). (C) anterior median dorsal plate of *Grossilepis rikiki* (IRSNB P.9254). (D) length vs. height graph of median dorsal plates of *Turrisaspis elektor* from Pennsylvania and *T*. *strudensis* from Belgium (number of specimens from Belgium non-statistically valid). The red dashed lines represent assumed limits between immature and adult specimens. (A)-(B) modified from [2], (C) modified from [4], (D) modified from [5].

A morphometric analysis of *Phyllolepis* [13] is used herein to characterize the ontogenetic composition of the *Phyllolepis* material from Strud (Fig 1A and 1B, S1 Table). The ontogenetic stages of *Grossilepis rikiki* are determined from morphological characteristics previously determined for immature bothriolepidids (Fig 1C). The material of *Turrisaspis strudensis* is compared to the more complete and extensive material of *Turrisaspis elektor* from Red Hill, Pennsylvania [25] to infer ontogenetic stages of the Belgian material (Fig 1D, S2 Table). In the case of small data sets, as is the case for the sample of anterior ventrolateral plates of *Phyllolepis* from Pennsylvania and the sample of median dorsal plates of *Turrisaspis strudensis* from Belgium, a test of significance for normality may not be sufficient to detect the deviation of the variable from normality.

The placoderm material from Belgium studied here is housed in the following Belgian institutions: Institut royal des Sciences naturelles de Belgique (IRSNB, Brussels), Université de Liège (ULg) and Université Catholique de Louvain-la-Neuve (UCL). The placoderm material from Pennsylvania is housed at the Academy of Natural Sciences of Philadelphia (ANSP), USA.

Specimen numbers are listed in S1 and S2 Tables. All specimens are accessible in permanent repositories of the above cited institutions. All necessary permits were obtained for the described study, which complied with all relevant regulations. The Gesves municipality, administrator of the Strud locality, gave us all authorizations for excavations and material collection.

## Results

The life cycle of a fish is divided into five ontogenetic stages: embryonic, larval, juvenile, adult and senescent [26–27]. Even in extant fishes, characters permitting the discrimination between these different stages are sometimes problematic. It is thus more complicated on fossil material, in which a large part of the morphological information is lost [20]. In this paper, the term “immature” is used to characterize embryonic, larval and juvenile stages, because discriminating stages in the fossil record is difficult [20], and size is used as a proxy for age [28] when morphological data do not permit assessment of the growth stage of our specimens.

Immature and adult stages are easily distinguishable in bothriolepidid antiarch placoderms because some sensory line grooves visible in immature forms are not visible on adults. In previous work, no precise growth studies have been performed on phyllolepidid and groenlandaspidid placoderms; it is therefore difficult, based on morphological characters, to distinguish immature and adult/senescent specimens in our material. Morphometric analyses on growth series can thus prove useful. In our study, upper size limits for immature specimens are assumed for *Phyllolepis undulata* and *Turrisaspis elektor* as made in previous studies [28].

### Antiarch placoderms

*Grossilepis rikiki* remains are extremely scarce at Strud; only four isolated bones have been found: two anterior median dorsal plates, one ventral central plate and one plate of the lateral marginal series of the pectoral appendage ([14], Fig 5B-D, F). Because *Grossilepis* is the sister group of *Bothriolepis* [29], it is assumed that both genera followed the same ontogenetic sequence. Immature characters that have clearly been identified for the anterior median dorsal plate of *Bothriolepis* [23,30,31] include: thin bone, nodose ornamentation, narrow shape, dorsal median ridge well-developed, presence of the anterior oblique dorsal sensory line grooves on the dorsal surface, posterior median process strongly developed, and well-pronounced fossae, grooves and thickenings in internal view. Both anterior median dorsal plates are thus interpreted as immature plates. The lateral spines of the lateral marginal plate 2 from Strud are numerous and sharp, and characterize immature material accordingly to a study on the ontogeny of the antiarch *Asterolepis* [21,22]. In addition, the nodose ornamentation of the ventral central plate 1 is also characteristic of immature material.

### Phyllolepidid placoderms

A recent morphometric analysis of centronuchal and anterior ventrolateral plates of *Phyllolepis* from different localities of Belgium and Pennsylvania demonstrated the presence of a single species at these Euramerican sites: *P*. *undulata* [13]. The cluster of plots of anterior ventrolateral plates of *Phyllolepis undulata* from Belgium (Fig 1A) along the lower part of the regression line indicates that a greater proportion of smaller individuals, interpreted as immature individuals, are present at Strud compared to larger individuals interpreted as adults. Addition of the specimens from Pennsylvania (Fig 1B), which show a uniform distribution, confirms the greater proportion of smaller individuals in Belgium and thus the greater proportion of immature individuals. It is assumed that small specimens (under a width of 26.5 mm) represent immature specimens, and large specimens (over a width of 26.5 mm) are considered to be adult.

### Groenlandaspidid placoderms

Specimens of *Turrisaspis elektor* from Red Hill, Pennsylvania, USA, have provided new information on the growth of the median dorsal plate of this taxon [25]. The lengths and widths of 29 median dorsal plates from this locality were plotted to clarify whether the variation in size and shape were due to interspecific, intraspecific and/or ontogenetic changes [25]. The continuous distribution argued for interpretation of the Red Hill sample as a single species with different ontogenetic stages [25]. In order to determine the ontogenetic nature of *Turrisaspis strudensis* material, its median dorsal plate measurements were plotted with those of the species from Pennsylvania (Fig 1D). The median dorsal plates from Strud plot along the lower part of the growth line, with the *T*. *elektor* specimens interpreted as being from immature individuals (specimens under a width of 20 mm are considered as immature). However, it does not necessarily mean that specimens from Strud are immature specimens as adult specimens of *T*. *strudensis* could be much smaller than the adults of *T*. *elektor*; this is the case for *Incisoscutum ritchiei* and *I*. *sarahae* [32].

Ontogenetic features for groenlandaspidid placoderms have never been the scope of a dedicated study, thus it is rather difficult to assign an ontogenetic stage to the few scattered remains from Strud. However, it was noticed for *Africanaspis doryssa* [33], that immature median dorsal plates were narrow (almost twice as high as long) and this is also observable for *Turrisaspis elektor* [25]. This character is recognized in the material from Strud, as such it is cautiously attributed to immature material.

## Discussion

### Reproductive strategies in placoderms

Placoderms had various reproductive strategies. Some placoderms gave birth to live young [34–36] whereas others laid eggs [19,37]. Neither eggs nor egg sacs have been recorded in Strud. The reproductive strategy of *Grossilepis* can be assumed, because internal fertilization has been suggested as the general mode of reproduction in Antiarchi [38]. No information is available regarding the reproductive strategy of *Turrisaspis*, nor that of other groenlandaspidid placoderms, despite internal fertilization and viviparity being known within various groups of arthrodires [39]. The reproductive strategy of *Phyllolepis* is also unknown, but there is evidence of internal fertilization in its close relative *Austrophyllolepis youngi* [35]. It cannot currently be determined whether *Phyllolepis* was an egg layer or gave live birth to live young, despite being a close relative of *Cowralepis mclachlani*, which seemed to have been an egg layer [37]. Although the present study does not bring information on the reproductive strategies of these vertebrates, it provides a better understanding of the postnatal strategies used by these three placoderm taxa.

### A placoderm nursery in Strud

Siliciclastic strata of the Strud quarry represent the filling of a channel in an alluvial plain [24]. The disarticulated nature of all vertebrate remains found at Strud suggests some post mortem transport. However, the excellent preservation of numerous small and fragile pieces, *e*.*g*. spinelets of the median dorsal plates of *Turrisaspis*, parasphenoid, basihyal and ceratohyals of *Phyllolepis* [13], argues for transport over a very short distance and a lack of reworking. The presence of rare adult placoderm remains and the abundance of large plant remains and large isolated sarcopterygian bones indicate the absence of size sorting. The effective presence, although very rare, of remains of adult placoderms also argues for an absence of taphonomic bias, because if there was sorting then adult and immature specimen remains would not co-occur. It was demonstrated that embryos and juveniles are often absent or underrepresented in a locality given the fragile nature of their dermal plates [40]. This argues for the fact that there were more immature specimens in the Strud ecosystem than estimated on the total number of collected fossil remains, which is in agreement with the nursery hypothesis. On the other hand, the sampling bias was strongly reduced, because Strud has been extensively excavated from 2004 to 2015 with all found placoderm dermal plates collected. Thus, the Late Devonian placoderm assemblage of the Strud locality likely represents a life assemblage. It is characterized by a placoderm community of nearly exclusively immature specimens and is here considered as a placoderm nursery.

Strud is not the first record of a placoderm nursery in the fossil record. The *Bothriolepis* nurseries noted from the Famennian of Tioga County, Pennsylvania [23] were interpreted as the hatchings from large numbers of eggs that were laid and then fertilized, although recent discoveries argue for internal fertilization in antiarchs [38], which produced eggs already fertilized in the case of the egg layer strategy. Moreover, Strud also represents the first occurrence of a placoderm nursery used at the same time by several placoderm taxa.

The Red Hill locality in Pennsylvania also produced *Turrisaspis* and *Phyllolepis* in association [25], but no antiarch remains were recovered. Contrary to observations made in Strud, placoderm material from Red Hill represents a wider range of ontogenetic stages ([13], fig 9; Fig 1B and 1D). The palaeoenvironment of Red Hill is interpreted as a meandering stream system with frequent avulsion events [41]. A complex depositional history of cut and fill may have reworked placoderm remains and mixed up ontogenetic stage distribution within the many facies represented there. Additionally, the steep outcrop at Red Hill does not often allow for excavation of single bedding planes, and thus the collection itself is an average of ontogenetic stages across the large site. For these reasons, no placoderm nursery pattern has ever been recognized in this locality.

**Fig 2:**
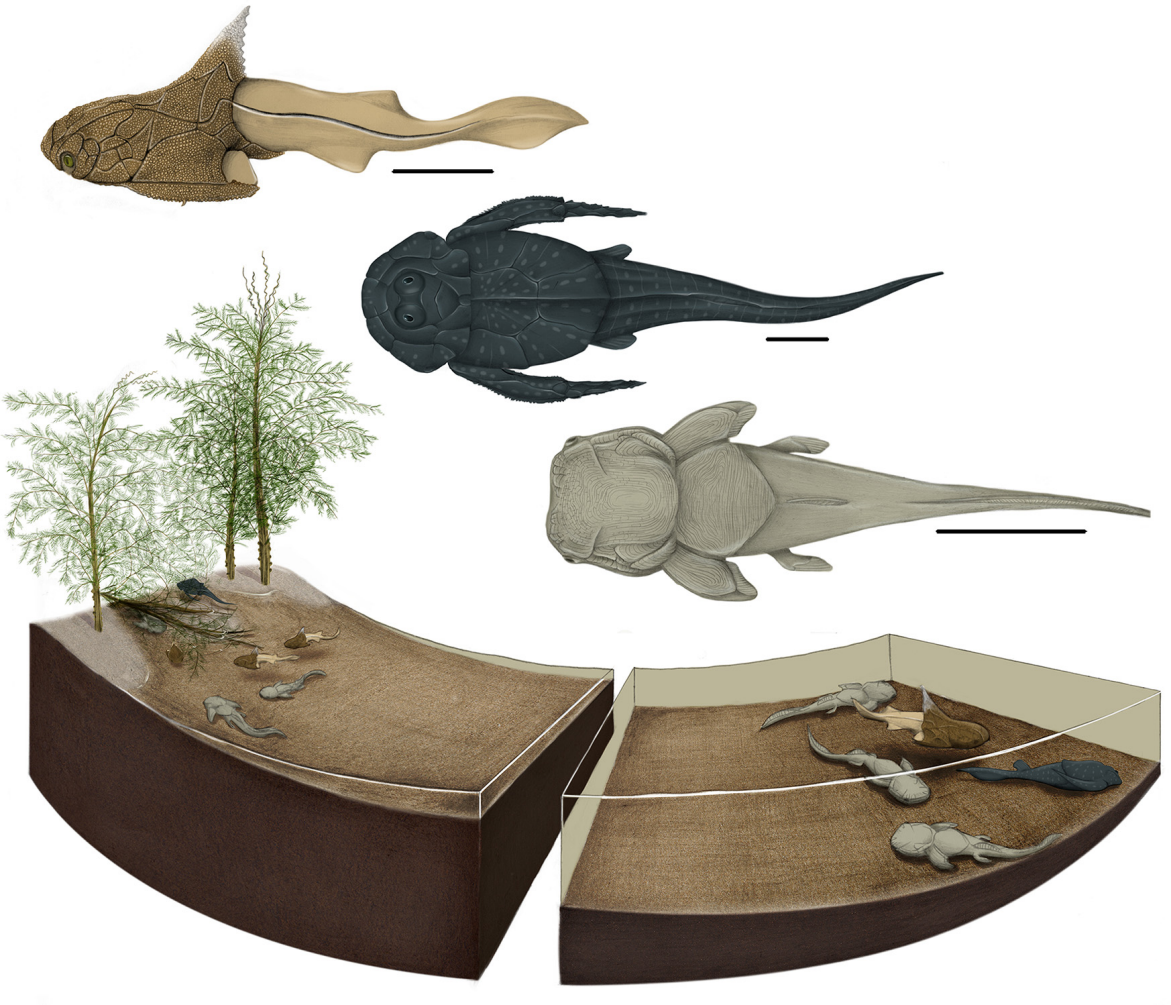
Reconstruction of the immature placoderms and diagrammatic model of the Strud nursery. Immature placoderms (from top to bottom) *Turrisaspis strudensis* (left lateral view), *Grossilepis rikiki* (dorsal view), *Phyllolepis undulata* (dorsal view). Diagrammatic model of the Strud nursery displaying the habitat partitioning: on the left, shallow waters of the nursery with immature placoderms inside and *Rhacophyton* plant on the bank; on the right, deeper area with the placoderm adults. Scale bars equal 2 cm. Animal and environmental reconstructions by J. Jacquot Haméon (MNHN, Paris).

The palaeoecology of the Strud nursery suggests a placoderm life history similar to that deduced from other fossil [23,42,43] and modern fishes [15], laying eggs or giving live birth in nearshore or in shallow continental environments. In those Devonian environments, shallow waters offer appropriate seasonal conditions with a minimized flow velocity [44] and could offer protection against large predators because of the numerous large, hard and sometimes spiny vegetal remains. The Strud nursery thus also implies the partitioning of the Strud placoderm habitat (Fig 2). A similar pattern was discussed for a freshwater Triassic selachian fauna [45]. Adult placoderms may have used the nursery of Strud only to lay eggs and/or give live birth and would have generally lived far from the nursery, in deeper waters.

## Supporting Information

S1 Table Anterior ventro-lateral plate length and width measurements of *Phyllolepis undulata* from Strud (Belgium) and Red Hill (USA). (PDF)

S2 Table Median dorsal plate height and length measurements of *Turrisaspis elektor* from Red Hill (USA) and *Turrisaspis strudensis* from Strud (Belgium). (PDF)
