# Supplementary material for: Placoderm Assemblage from the Tetrapod-Bearing Locality of Strud (Belgium, Upper Famennian) Provides Evidence for a Fish Nursery
Source: PLoS One. 2016 Aug 23;11(8):e0161540. doi: 10.1371/journal.pone.0161540 (PMC4994939; doi:10.1371/journal.pone.0161540)
Supplement: S1 Table — (PDF) [file pone.0161540.s001.pdf]

Collection number	Length (mm)	Width (mm)	Taxon	Locality	Remarks
IRSNB vert 31.264-031	23	24	*Phyllolepis undulata*	Strud, Belgium	
IRSNB vert 31.264-032	20	18	*Phyllolepis undulata*	Strud, Belgium	
IRSNB vert 32.164-011	25	21	*Phyllolepis undulata*	Strud, Belgium	
IRSNB vert 32.220-035	13	10	*Phyllolepis undulata*	Strud, Belgium	
IRSNB P.9476	11	10	*Phyllolepis undulata*	Strud, Belgium	
IRSNB vert 31.264-033	21	17	*Phyllolepis undulata*	Strud, Belgium	
IRSNB vert 32.220-036	22	19	*Phyllolepis undulata*	Strud, Belgium	
IRSNB vert 31.264-034	14	14	*Phyllolepis undulata*	Strud, Belgium	
IRSNB vert 31.913-010	20	18	*Phyllolepis undulata*	Strud, Belgium	
IRSNB vert 31.913-011	24	20	*Phyllolepis undulata*	Strud, Belgium	
IRSNB vert 31.595-010	35	30	*Phyllolepis undulata*	Strud, Belgium	
IRSNB vert 31.913-012	14	11	*Phyllolepis undulata*	Strud, Belgium	
IRSNB vert 31.264-036	23	22	*Phyllolepis undulata*	Strud, Belgium	
IRSNB vert 32.048-002	20	15	*Phyllolepis undulata*	Strud, Belgium	
IRSNB vert 31.913-014	18	15	*Phyllolepis undulata*	Strud, Belgium	
IRSNB P.9477	21	20	*Phyllolepis undulata*	Strud, Belgium	
IRSNB vert 31.264-038	26	24	*Phyllolepis undulata*	Strud, Belgium	
IRSNB P.9478	49	43	*Phyllolepis undulata*	Strud, Belgium	
IRSNB vert 31.264-039	16	15	*Phyllolepis undulata*	Strud, Belgium	
IRSNB vert 31.913-015	11	10	*Phyllolepis undulata*	Strud, Belgium	
IRSNB vert 31.264-041	24	22	*Phyllolepis undulata*	Strud, Belgium	
IRSNB vert 31.264-042	34	27	*Phyllolepis undulata*	Strud, Belgium	
IRSNB vert 31.264-043	22	16	*Phyllolepis undulata*	Strud, Belgium	

Collection number	Length (mm)	Width (mm)	Taxon	Locality	Remarks
IRSNB vert 31.264-044	23	18	*Phyllolepis undulata*	Strud, Belgium	
IRSNB vert 31.264-045	18	16	*Phyllolepis undulata*	Strud, Belgium	
IRSNB vert 32.164-012	17	15	*Phyllolepis undulata*	Strud, Belgium	
IRSNB P.9479	16	14	*Phyllolepis undulata*	Strud, Belgium	
IRSNB vert 32.164-013	17	14	*Phyllolepis undulata*	Strud, Belgium	
IRSNB P.9480	13	12	*Phyllolepis undulata*	Strud, Belgium	
IRSNB vert 31.264-046	19	14	*Phyllolepis undulata*	Strud, Belgium	
IRSNB P.9481	40	33	*Phyllolepis undulata*	Strud, Belgium	
IRSNB vert. 31.264-047	27	22	*Phyllolepis undulata*	Strud, Belgium	
IRSNB vert 31.264-048	26	23	*Phyllolepis undulata*	Strud, Belgium	
IRSNB vert 31.264-049	18	15	*Phyllolepis undulata*	Strud, Belgium	
IRSNB vert 31.595-011	18	15	*Phyllolepis undulata*	Strud, Belgium	
IRSNB vert 32.438-027	10	10	*Phyllolepis undulata*	Strud, Belgium	
IRSNB vert 32.438-028	26	26	*Phyllolepis undulata*	Strud, Belgium	
IRSNB vert 32.438-029	15	15	*Phyllolepis undulata*	Strud, Belgium	
IRSNB P.9482	27	25	*Phyllolepis undulata*	Strud, Belgium	
IRSNB vert 32.438-030	33	28	*Phyllolepis undulata*	Strud, Belgium	
IRSNB vert 32.438-031	12	10	*Phyllolepis undulata*	Strud, Belgium	
IRSNB vert 32.438-032	20	17	*Phyllolepis undulata*	Strud, Belgium	
PALULG 2013. 05.03.11	16	14	*Phyllolepis undulata*	Strud, Belgium	
PALULG.2014. 04.08.16	14	13	*Phyllolepis undulata*	Strud, Belgium	
PALULG.2014. 04.08.15	26	21	*Phyllolepis undulata*	Strud, Belgium	
?	25	23	*Phyllolepis undulata*	Strud, Belgium	Specimen not found. Measurements according to [10], pl.11, fig. 3

Collection number	Length (mm)	Width (mm)	Taxon	Locality	Remarks
?	19	15	*Phyllolepis undulata*	Strud, Belgium	Specimen not found. Measurements according to [10], pl.11, fig. 5
?	20	17	*Phyllolepis undulata*	Strud, Belgium	Specimen not found. Measurements according to [10], pl.11, fig. 6
?	37	38	*Phyllolepis undulata*	Chèvremont, Belgium	Specimen not found. Measurements according to [10], pl.11, fig. 4
PALULG.2013.05.03.11	15	15	*Phyllolepis undulata*	Chèvremont, Belgium	
PALULG.2014.01.29.16	28	23	*Phyllolepis undulata*	Chèvremont, Belgium	
PALULG.2014.01.29.19a	20	17	*Phyllolepis undulata*	Chèvremont, Belgium	
PALULG.2014.01.29.19b	23	19	*Phyllolepis undulata*	Chèvremont, Belgium	
ANSP 20826	36	29	*Phyllolepis undulata*	Red Hill, USA	
ANSP 20818	20	16	*Phyllolepis undulata*	Red Hill, USA	
ANSP 20827	27	24	*Phyllolepis undulata*	Red Hill, USA	
ANSP 20822	48	40	*Phyllolepis undulata*	Red Hill, USA	
ANSP 21185	52	43	*Phyllolepis undulata*	Red Hill, USA	
ANSP 20823	50	48	*Phyllolepis undulata*	Red Hill, USA	
ANSP 23396	36	30	*Phyllolepis undulata*	Red Hill, USA	
ANSP 20821	50	38	*Phyllolepis undulata*	Red Hill, USA	
ANSP 20824	30	30	*Phyllolepis undulata*	Red Hill, USA	
