# Supplementary material for: Placoderm Assemblage from the Tetrapod-Bearing Locality of Strud (Belgium, Upper Famennian) Provides Evidence for a Fish Nursery
Source: PLoS One. 2016 Aug 23;11(8):e0161540. doi: 10.1371/journal.pone.0161540 (PMC4994939; doi:10.1371/journal.pone.0161540)
Supplement: S2 Table — (PDF) [file pone.0161540.s002.pdf]

Collection number	Length (mm)	Height (mm)	Taxon	Locality
ANSP 20669	9.7	23.1	*Turrisaspis elektor*	Red Hill, USA
ANSP 20674	8.4	12.8	*Turrisaspis elektor*	Red Hill, USA
ANSP 20678	6.1	13.8	*Turrisaspis elektor*	Red Hill, USA
ANSP 20687	17.6	31.1	*Turrisaspis elektor*	Red Hill, USA
ANSP 20703	17.6	28.2	*Turrisaspis elektor*	Red Hill, USA
ANSP 20707	7.6	14.2	*Turrisaspis elektor*	Red Hill, USA
ANSP 20708	6.3	10.7	*Turrisaspis elektor*	Red Hill, USA
ANSP 20711	14.5	24.7	*Turrisaspis elektor*	Red Hill, USA
ANSP 20713	26.2	30.1	*Turrisaspis elektor*	Red Hill, USA
ANSP 20714	18.5	28.5	*Turrisaspis elektor*	Red Hill, USA
ANSP 20715	22.5	29.2	*Turrisaspis elektor*	Red Hill, USA
ANSP 20721	5.9	10.2	*Turrisaspis elektor*	Red Hill, USA
ANSP 20731	9	10.8	*Turrisaspis elektor*	Red Hill, USA
ANSP 20736	12.2	21.4	*Turrisaspis elektor*	Red Hill, USA
ANSP 20756	16	21.6	*Turrisaspis elektor*	Red Hill, USA
ANSP 20757	11.7	20.7	*Turrisaspis elektor*	Red Hill, USA
ANSP 20759	17.9	26.6	*Turrisaspis elektor*	Red Hill, USA
ANSP 20786	7.1	14.4	*Turrisaspis elektor*	Red Hill, USA

Collection number	Length (mm)	Height (mm)	Taxon	Locality
ANSP 20787	28.1	28.5	*Turrisaspis elektor*	Red Hill, USA
ANSP 20790	14.9	23.2	*Turrisaspis elektor*	Red Hill, USA
ANSP 20797	15.4	23.2	*Turrisaspis elektor*	Red Hill, USA
ANSP 20936	8.1	13.9	*Turrisaspis elektor*	Red Hill, USA
ANSP 20939	17.6	22	*Turrisaspis elektor*	Red Hill, USA
ANSP 20944	5.3	6.9	*Turrisaspis elektor*	Red Hill, USA
ANSP 20949	11.5	22.7	*Turrisaspis elektor*	Red Hill, USA
ANSP 20961	15.5	16.7	*Turrisaspis elektor*	Red Hill, USA
ANSP 21297	19	28.7	*Turrisaspis elektor*	Red Hill, USA
ANSP 21301	17.7	25.7	*Turrisaspis elektor*	Red Hill, USA
ANSP 21325	12.2	23.7	*Turrisaspis elektor*	Red Hill, USA
UCL PVL 10.532	12.8	16	*Turrisaspis strudensis*	Strud, Belgium
IRSNB P.9449	11.2	16.4	*Turrisaspis strudensis*	Strud, Belgium
IRSNB P.9450	10	16	*Turrisaspis strudensis*	Strud, Belgium
